# CircGFRA1 facilitates the malignant progression of HER‐2‐positive breast cancer via acting as a sponge of miR‐1228 and enhancing AIFM2 expression

**DOI:** 10.1111/jcmm.16963

**Published:** 2021-10-20

**Authors:** Meiheban Bazhabayi, Xingsheng Qiu, Xing Li, Anli Yang, Wei Wen, Xiaoli Zhang, Xiangsheng Xiao, Rongfang He, Peng Liu

**Affiliations:** ^1^ Department of Breast Oncology Sun Yat‐Sen University Cancer Center State Key Laboratory of Oncology in South China Collaborative Innovation Center for Cancer Medicine Guangzhou China; ^2^ Department of Radiation Oncology Sun Yat‐Sen Memorial Hospital Sun Yat‐Sen University Guangzhou China; ^3^ Department of Pathology The First Affiliated Hospital Hengyang Medical School University of South China Hengyang China

**Keywords:** AIFM2, circGFRA1, ferroptosis, HER‐2‐positive breast cancer, miR‐1228

## Abstract

CircRNAs (circular RNA) are reported to regulate onset and progress multiple cancers. Nonetheless, the function along with the underlying mechanisms of circRNAs in HER‐2‐positive breast cancer (BC) remains unclear. CircRNA microarrays were performed to elucidate expression profiles of HER‐2‐positive BC cells. circRNA levels were quantified using qRT‐PCR assay. Various in vitro along with in vivo assays were employed to further explore the effects of circGFRA1 in the progress of HER‐2‐positive BC and interactions of circGFRA1, miR‐1228 and AIFM2 in Her‐2‐positive BC. CircGFRA1 was remarkably upregulated in HER‐2‐positive BC. Knockdown of circGFRA1 could attenuate HER‐2‐positive BC progression by inhibiting the proliferation, infiltration and migratory ability of HER‐2‐positive BC cells. Through ceRNA mechanism, circGFRA1 could bind to miR‐1228 and alleviate inhibitory activity of miR‐1228 on targeted gene AIFM2. In summary, circGFRA1‐miR‐1228‐AIFM2 axis regulates HER‐2‐positive BC. CircGFRA1 is a novel promising treatment option for HER‐2‐positive BC.

## 1 INTRODUCTION

Breast cancer (BC) is considered a complex carcinoma that affects many women around the world. BC constitute a major cause of death in women, at a proportion of 11% of all types of cancer diagnosed worldwide each year.^1^ According to immunohistochemical markers, BC is clinically divided into the following three main subtypes to improve targeted therapy: ER (oestrogen receptor)‐positive, TNBC (triple‐negative) BC and HER‐2 (human epidermal growth factor receptor 2)‐positive.^2^ HER‐2‐positive tumours represent 20% of BCs.^3^ Tumours with elevated HER‐2 levels grow at a higher rate and are more aggressive; therefore, the HER‐2 gene plays an important role in the tumour biological process.^4^ Thus, the development of more effective therapeutic strategies is crucial to improve the survival of individuals with HER‐2‐positive BC.

CircRNA research has gradually become one of the most compelling research area due to its connection with human disease, especially with cancer.^5^, ^6^, ^7^, ^8^, ^9^ CircRNAs (circular RNAs) are derived from back‐splicing circularization of pre‐mRNAs (precursor mRNAs). They are single‐stranded, stable covalently closed‐loop structures that lack accessible 3′ or 5′ ends.^10^, ^11^ CircRNAs are able to regulate translation, bind to proteins and acting as miRNA sponges of miRNA competitive endogenous RNAs (ceRNAs).^12^ In the context of cancer, circRNAs have been shown to act as repressors or promoters of tumour biological processes.^13^, ^14^ For example, circFBXW7 suppresses the malignancy of TNBC (triple‐negative BC) by inhibiting TNBC growth and migration.^13^ Circ‐ZNF609 acts as an oncogenic circRNA that promotes renal carcinoma cell proliferation and invasion.^14^ However, the role of circRNAs in HER‐2‐positive BC remains ambiguous.

MiRNAs (microRNAs) are small, noncoding RNAs with a length of 22 nt. As negative modulators of mRNA (messenger RNA), miRNA can decrease the stability or limit the translation of mRNA by pairing with docking sites on the 3′‐UTR (3′‐untranslated region) on specific mRNAs.^15^ In malignant tissues, miRNAs that are upregulated and promote tumour cell proliferation, invasion and apoptosis resistance are known as oncomiRs. Conversely, miRNAs that are downregulated and exhibit the opposite properties of oncomiRs described above are called tumour suppressor miRs.^16^, ^17^ Recent studies have demonstrated that circRNAs with ceRNA or miRNA sponge activity bind to miRNAs via MREs (miRNA response elements) and relieve the miRNA repressive influence of miRNAs on their target mRNAs.^18^, ^19^ For example, in gastric cancer (GC), circCCDC9 inhibits the aggressive biological nature of GC cells by sponging miR‐6792‐3p.^20^ Circ‐ADAM9 enhances the proliferation, infiltration along with migration characteristics of pancreatic cancer cells through working as a sponge for miR‐127.^21^ Nevertheless, if the circRNA‐miRNA modulatory network participates in the progress of HER‐2‐positive BC is elusive.

Here, we identified a type of HER‐2‐positive BC‐related circRNA, hsa_circ_005239, which is predicted to be originated from the GFRA1 (GDNF family receptor alpha1) gene. Hence, we termed hsa_circ_005239 circGFRA1. To gain deeper insight into the clinical significance of circGFRA1 in HER‐2‐positive BC, the role and potential mechanism of circGFRA1 in the biological process of HER‐2 positive BC were explored. CircGFRA1 was upregulated in HER‐2‐positive BC and sponged miR‐1228 to affect AIFM2 expression and therefore modulate the onset and progress HER‐2‐positive BC.

## 2 MATERIALS AND METHODS

### 2.1 Patients samples and ethical standards

Fresh primary breast cancer tissues were collected from Sun Yat‐Sen University Cancer Center (SYSUCC, Guangzhou, Guangdong, China), State Key Laboratory of Oncology in South China, and were frozen in liquid nitrogen immediately after resection. Patients receiving neoadjuvant therapy were excluded. This study was approved by the Ethics Committee of the SYSUCC and performed in accordance with the Declaration of Helsinki. Written informed consent was obtained from all patients before participation in this study.

### 2.2 Cell culture and transfection

The HME cell line (184A) along with the human HER‐2‐positive BC cells (SKBR3 and BT474) was supplied by ATCC (MD, USA). All cells were passaged for≤6 months and cultured properly according to the instructions. Mycoplasma infection was not detected in the cells, and the authenticity of the cells was proven by DNA fingerprinting.

The Lipofectamine 2000 platform (Cat No, Invitrogen) was adopted for cell transfections. siRNAs that targeted circGFRA1 were supplied by GenePharma. The siRNA sequences utilized in this study are shown in Table 1. MiR‐1228 mimics were designed and synthesized by GeneCopoeia.

**TABLE 1:** The target sequences of siRNAs used in this study

siRNA	Species	Target sequences
si‐control	Human	UUCUCCGAACGUGUCACGUTT
si‐circGFRA1#1	Human	GCAAATTTACAGATCTCGCCT
si‐circGFRA1#2	Human	TACAGATCTCGCCTTGCGGAT

### 2.3 Quantitative real‐time PCR (qRT‐PCR)

Extraction of total RNA was performed by TRIzol reagent (Cat No, Invitrogen). Isolation of cytoplasmic and nuclear RNAs was conducted with the NE‐PER Nuclear and Cytoplasmic Extraction Reagents (Thermo Scientific). After that, SYBR Premix Ex Taq (Cat No, Takara, Japan) along with All‐in‐OneTM miRNA qRT‐PCR Detection Kit (Cat No, GeneCopoeia) was employed to perform qRT‐PCR. The primers are shown in Table 2.

**TABLE 2:** Primer sequences for qRT‐PCRs used in this study

	Species	Direction	Sequence (5′ ‐ 3′)
18S	Human	Forward	AACTGGAATCGCATCAGGAC
		Reverse	AGGAGCTGCTCTGGGTGTAA
circGFRA1	Human	Forward	CCCTCCGGGTTAAGAACAAG
		Reverse	GGCAGTCAGCGTAGTTTTCC
hsa‐miR−1228	Human	Forward	ACACTCCAGCTGGG
		Reverse	TCACACCTGCCTCG
AIFM2	Human	Forward	AGACAGGGTTCGCCAAAAAGA
		Reverse	CAGGTCTATCCCCACTACTAGC
GAPDH	Human	Forward	GGAGCGAGATCCCTCCAAAAT
		Reverse	GGCTGTTGTCATACTTCTCATGG
β‐actin	Human	Forward	AGCGAGCATCCCCCAAAGTT
		Reverse	GGGCACGAAGGCTCATCATT

### 2.4 RNase R digestion assay

After isolation from SKBR3 cells, RNAs samples were assigned to two groups: the first group for RNase R (Epicentre Technologies) treatment and the second group was treated with buffer as a control. 2 µg of total RNA was inoculated at 37°C with RNase R (3 U/µg) for 20 min in the experimental group, with β‐Actin serving as the standard.

### 2.5 Actinomycin D assay

1 × 10^5^ cells were inoculated into 6‐well plates, and after treatment with actinomycin D (2 mg/L; Cat No, Sigma), we collected the treated cells at the indicated times (8 h, 16 h, 24 h) for qRT‐PCR evaluation of circGFRA1 and GFRA1 mRNA.

### 2.6 CCK‐8 assay

A CCK‐8 assay was conducted to evaluate cell proliferation. 1 × 10^3^ cells were inoculated into 96‐well plates and transfected. Then, added 10 µL of CCK‐8 solution (Cat No, Dojindo) 48 h post‐transfection. After 2 h in an incubator at 37°C, the absorbance was measured at 450 nM with a microtiter plate reader (Bio‐Tek EPOCH2).

### 2.7 Transwell assay

The invasion test was performed in migration chambers (BD Biosciences) to which a total of 1 × 10^4^ cells were introduced. After that, 10% FBS was introduced as an attractant into the lower compartment and left for 24 h in an incubator at 37°C. The cells were then fixed (with methanol), followed by staining (in 0.1% crystal violet) and finally counted.

### 2.8 Mouse xenograft model

Animal Experimental Ethics Committee of SYSUCC of our institution approved the study. Female BALB/c nude mice aged 4 weeks were administered subcutaneously with 2 × 10^6^ cells (five mice/group). After that, the mice were given intratumoural inoculation of 40 μL si‐circGFRA1 or si‐NC every 4 days. We euthanized the mice at 4 weeks following injection and determined the tumour weights.

To detect lung metastases, we intravenously inoculated 1 × 10^5^ cells into the tail veins of mice (six mice/group). After the elapse of 8 8 weeks, we anaesthetized the mice, harvested their lungs and visually counted the metastatic nodules in the lungs, followed by validation via H&E staining and counting under a microscope.

### 2.9 Luciferase reporter assay

5 × 10^3^ cells were planted in plates and cotransfected with the created vectors and miR‐1228 mimics for 48 h. Thereafter, a dual‐luciferase reporter assay kit (Cat No, Promega) was employed to explore the relative luciferase enzyme activity.

### 2.10 RIP assay

MS2bs‐circGFRA1, MS2bs‐circGFRA1mt or MS2bs‐Rluc were employed to cotransfect cells and incubated for 48 h. Afterwards, the Magna RIP RNA‐Binding Protein Immunoprecipitation Kit (Cat No, Millipore, USA) was employed to perform the RIP assay. Then, we purified the RNA complexes and quantitated the miR‐1228 content.

An anti‐Ago2 antibody (Cat No, Millipore) was adopted to perform the Ago2‐RIP assay, followed by RNA purification. Thereafter, quantification of the circGFRA1, AIFM2 and miR‐1228 contents was done.

### 2.11 Western blots

SDS‐PAGE (10%) was used to extract and separate total proteins, which were transfer‐embedded onto PVDF membranes (Cat No, Millipore, USA). Blocking of membranes was performed at room temperature using 5% skimmed milk for 1 h. Afterwards, the membranes were inoculated with primary anti‐AIFM2 (Cat No, Abcam; 1:100), anti‐GPX4 (Cat No, Abcam; 1:100) or anti‐β‐actin (Affinity, United States; 1:1000) antibodies. Subsequently, the membranes were inoculated with the HRP‐labelled secondary antibody (CST) and chemiluminescence employed to detect the bands.

### 2.12 Evaluation of GSH/GSSG ratio

The intracellular ratio of GSH (glutathione) and oxidized glutathione (GSSG) levels was determined by using the GSH/GSSG Quantification Kit II (Cat No, Dojindo) as described by the manufacturer, including preparation of sample and Standard Solution, and detection of concentration. The relative levels were analysed on a microplate reader (Molecular Devices Flexstation3).

## 3 RESULTS

### 3.1 CircGFRA1 is upregulated in HER‐2‐positive BC cells and tissues

**FIGURE 1:**
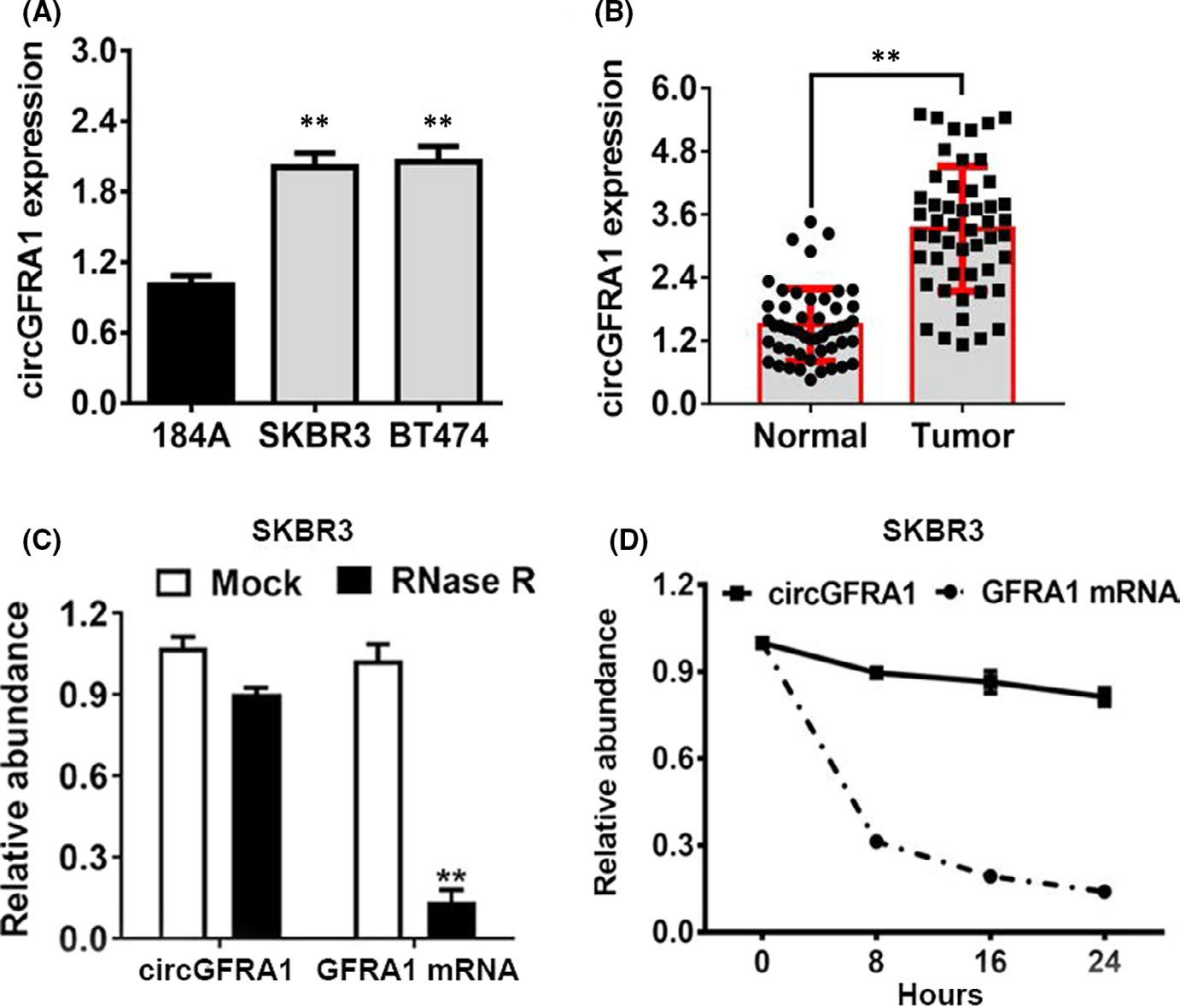
CircGFRA1 is upregulated in HER‐2‐positive BC cells and tissues. (A) The circGFRA1 content in HER‐2‐positive BC cell lines. (B) The circGFRA1 expression level in 50 pairs of HER‐2‐positive BC tissues and paired non‐malignant tissues. (C) RNase R degradation assay was employed to verify the circular nature of circGFRA1 in SKBR3 cell line. (D) Actinomycin D assays of the stability of the circular circGFRA1, as well as linear GFRA1 transcripts in SW620 cells

The circGFRA1 expression level in an HME (human mammary epithelial) cell line (184A) and two HER‐2‐positive BC cells (SKBR3 and BT474) was examined. The graph shows that circGFRA1 was upregulated in SKBR3, as well as BT474 cells (Figure 1A). In addition, in contrast with that in paired non‐malignant tissues, we found that circGFRA1 level was elevated in HER‐2‐positive BC tissues, as illustrated in Figure 1B. Subsequently, an RNase R degradation assay was carried out to verify the circular nature of circGFRA1 (Figure 1C). In addition, we adopted the actinomycin D test, which revealed that circGFRA1 mRNA showed higher stability in contrast with linear GFRA1 transcript in SW620 cells (Figure 1D).

### 3.2 Silencing circGFRA1 suppresses the proliferation of HER‐2‐positive BC

**FIGURE 2:**
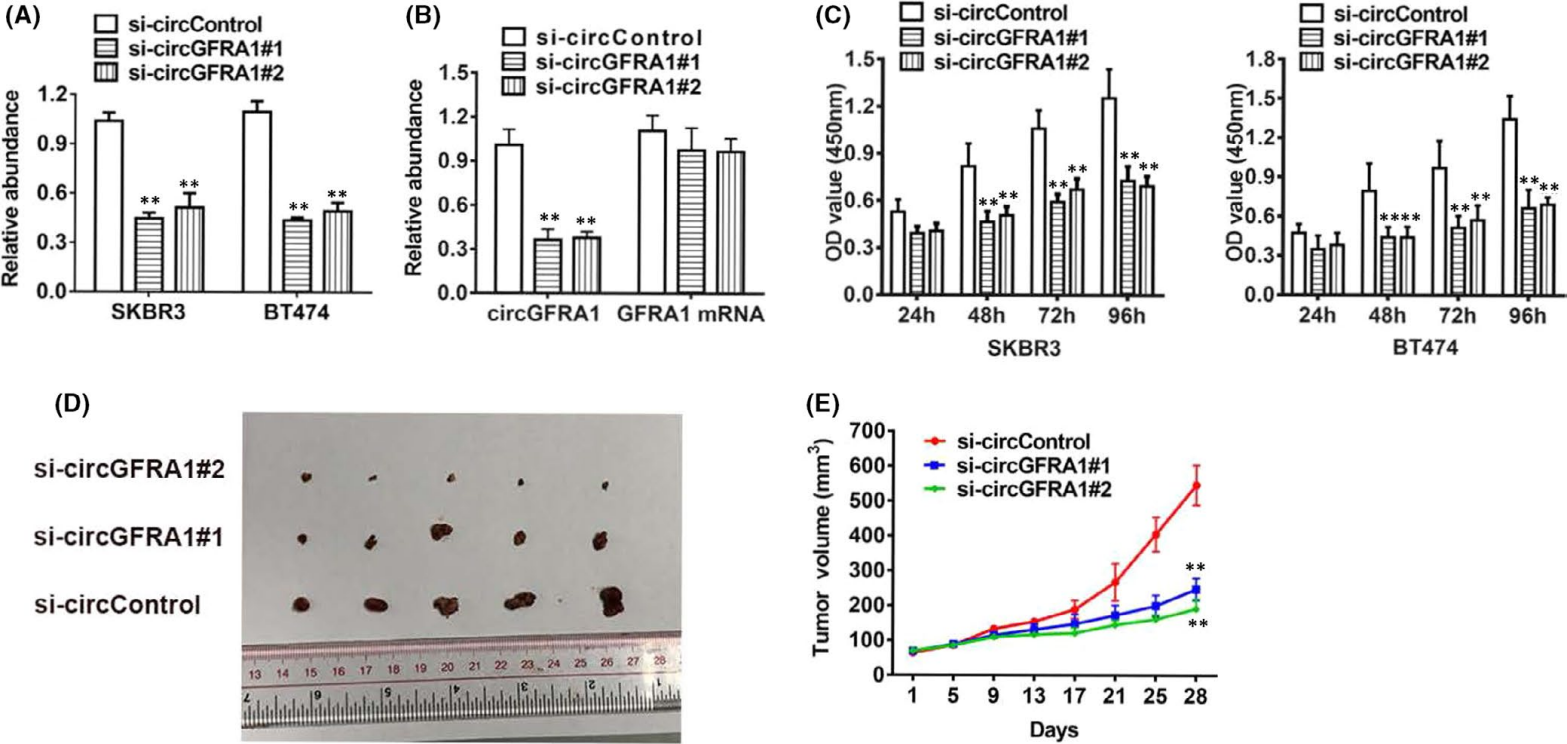
Knockdown of circGFRA1 suppresses the proliferation of HER‐2‐positive BC. (A) circGFRA1#1 successfully knocked down circGFRA1. (B) Loss‐of‐function assay to knock down circGFRA1 and GFRA1 mRNA. (C) CCK‐8 assay of cell proliferation. (D) Xenograft models were created. (E) Tumour volume was assessed every 4 days for 4 weeks

To investigate whether circGFRA1 is involved in HER‐2‐positive BC cell proliferation, we conducted loss‐of‐function assays. Two different siRNAs were designed to silence circGFRA1 through targeting the back‐splicing site. After transfection with the siRNAs, the expression level of circGFRA1 was reduced (Figure 2A), while no influence was observed on linear GFRA1 mRNA expression (Figure 2B). CCK‐8 assays illustrated that downregulation of circGFRA1 repressed cell proliferation (Figure 2C). In addition, we created mice xenograft models to help to determine the in vivo effects of circGFRA1, and various tumour volumes determined at diverse time points illustrated that depletion of circGFRA1 could retard tumour growth, as illustrated in Figure 2D,E.

### 3.3 Knockdown of circGFRA1 represses infiltration and metastasis of HER‐2‐positive BC

**FIGURE 3:**
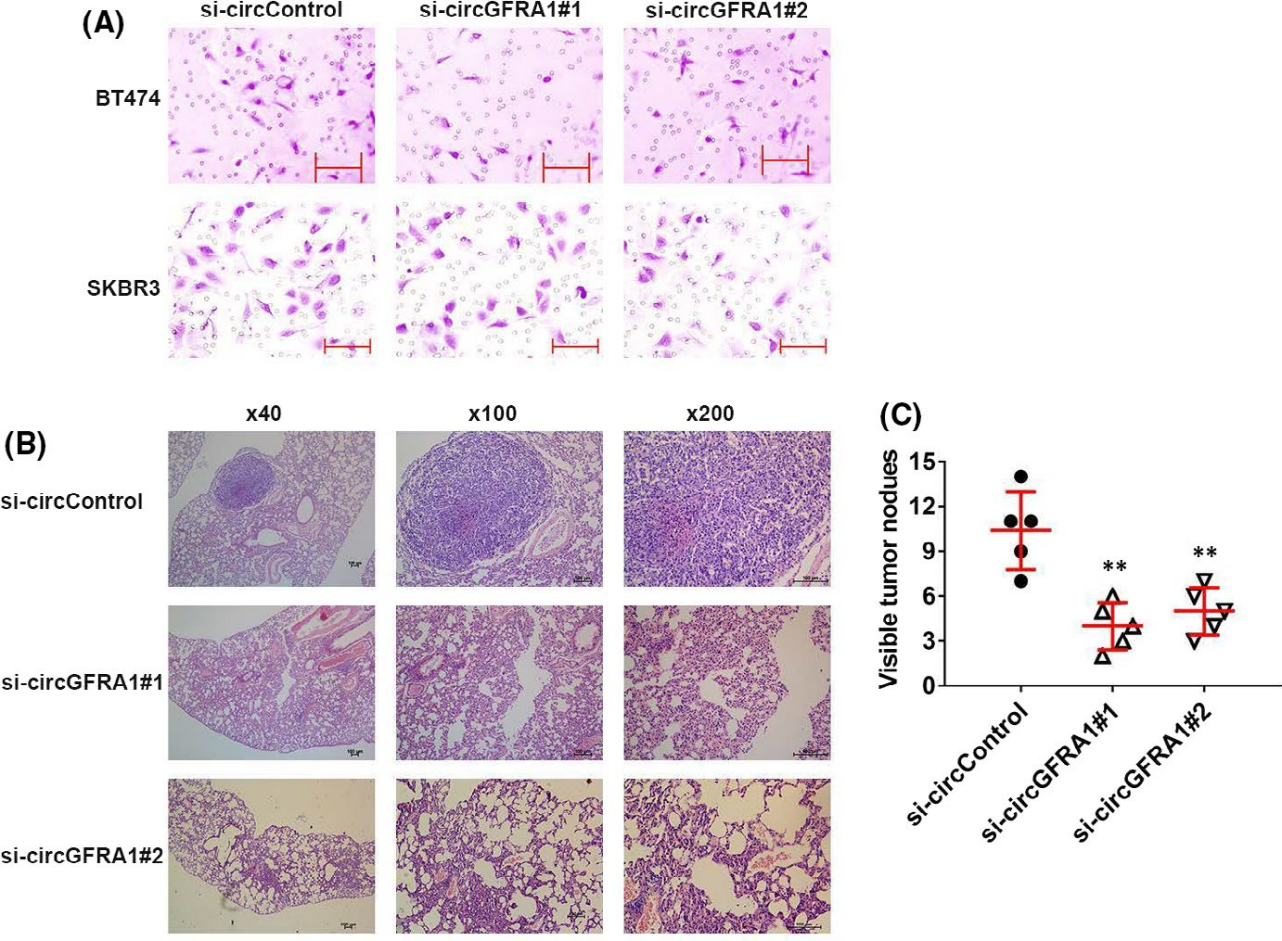
Downregulation of circGFRA1 represses the infiltration and metastasis in HER‐2‐positive BC. (A) Transwell assay of the infiltration ability of HER‐2‐positive BC cells. (B) H&E‐stained lung metastatic nodule sections. (C) Quantification of lung metastatic nodules

To assess the effect of circGFRA1 on the infiltration of HER‐2‐positive BC cells, a Transwell assay was performed. The results showed that circGFRA1 silencing could remarkably inhibit the percentage of invasive SKBR3 and BT474 cells (Figure 3A). Additionally, this result could also be observed in lung metastasis experiments in vivo. Suppression of circGFRA1 could decrease the number of lung metastases, illustrating that circGFRA1 is a significant modulator of the metastatic ability of HER‐2‐positive BC (Figure 3B,C).

### 3.4 CircGFRA1 acts as a sponge of miR‐1228

**FIGURE 4:**
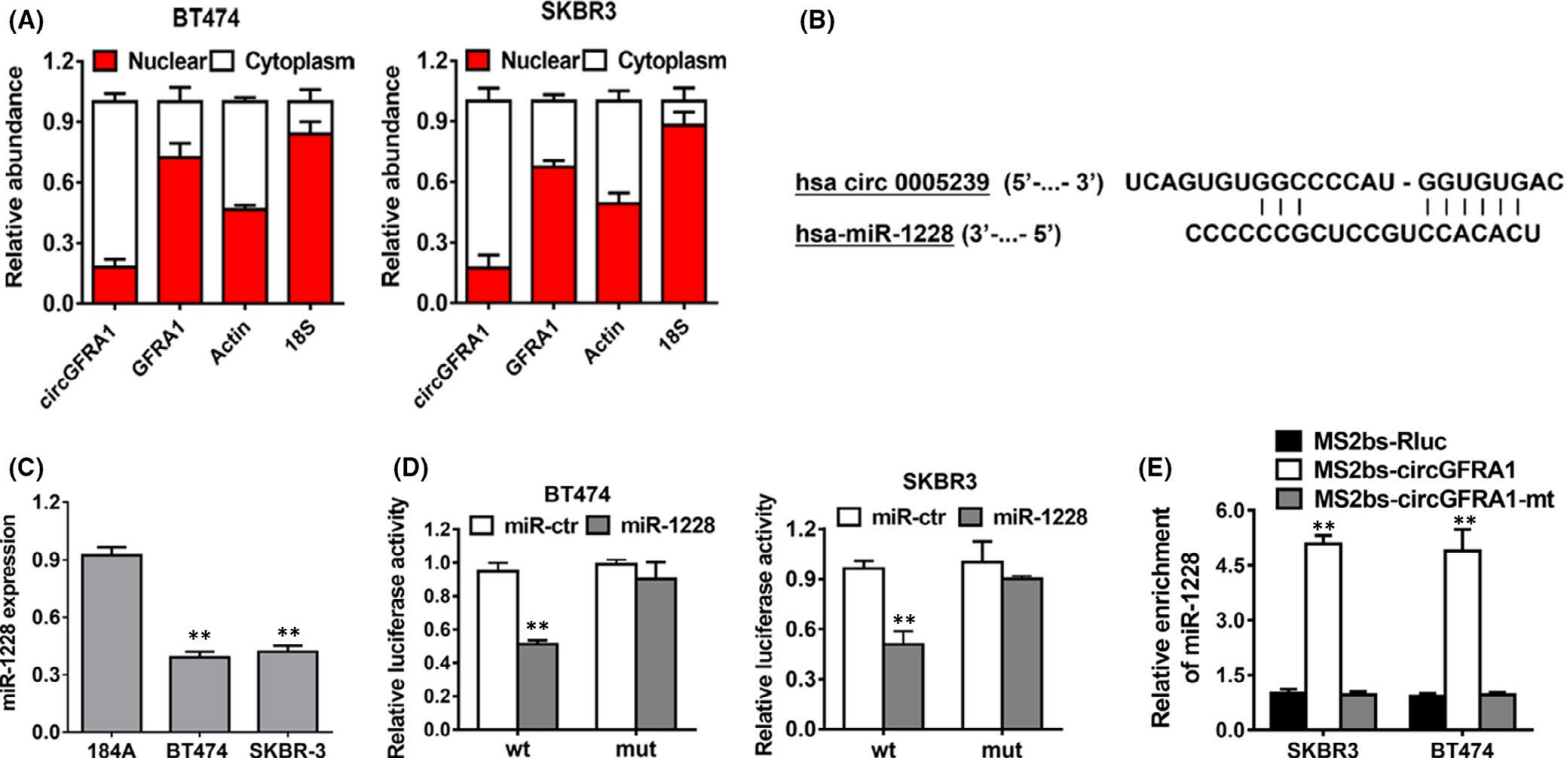
CircGFRA1 acts as a sponge of miR‐1228. (A) The relative levels of circGFRA1, GFRA1, β‐actin (cytoplasmic control) and 18S (nuclear control) were examined. (B) The predicted direct docking sites of miR‐128 within the circGFRA1 sequence. (C) The miR‐1228 content in the HME (184A) cell line, as well asHER‐2‐positive BC cell lines. (D) The relative luciferase activity of BT474 (left) and SKBR3 (right) cells cotransfected with the miR‐1228 mimics and the wild‐type or mutant luciferase enzyme reporter of circGFRA1. (E) MS2‐based RIP assay in HER‐2‐positive BC cell transfects of MS2bs‐circGFRA1, MS2bs‐circGFRA1‐mt or MS2bs‐Rluc

Further experiments determined that circGFRA1 predominantly existed in the cytoplasm, which indicates that it could crosstalk with miRNAs, which are mostly located in the cytoplasm (Figure 4A). Subsequently, we predicted the possible molecular target of circGFRA1 on the basis of the circRNA interactome (https://circinteractome.nia.nih.gov). As the outcome suggests, miR‐1228 has the potential to interact with circGFRA1 (Figure 4B). Next, we uncovered that miR‐1228 was decreased in HER‐2‐positive BC cells (Figure 4C). Additionally, a luciferase enzyme reporter assay data demonstrated that in the cells transfects of the wild‐type luciferase reporter and miR‐1228 mimics, the luciferase enzyme activity was repressed; nonetheless, the mutant luciferase enzyme reporter had no such impact (Figure 4D). RNA immunoprecipitation (RIP) assays illustrated that miR‐1228 was majorly abundant in the MS2bs‐circGFRA1 group (Figure 4E), which indicated a direct crosstalk between circGFRA1 and miR‐1228. Overall, circGFRA1 sponges miR‐1228.

### 3.5 Downregulation of circGFRA1 promotes ferroptosis in HER‐2‐positive BC

**FIGURE 5:**
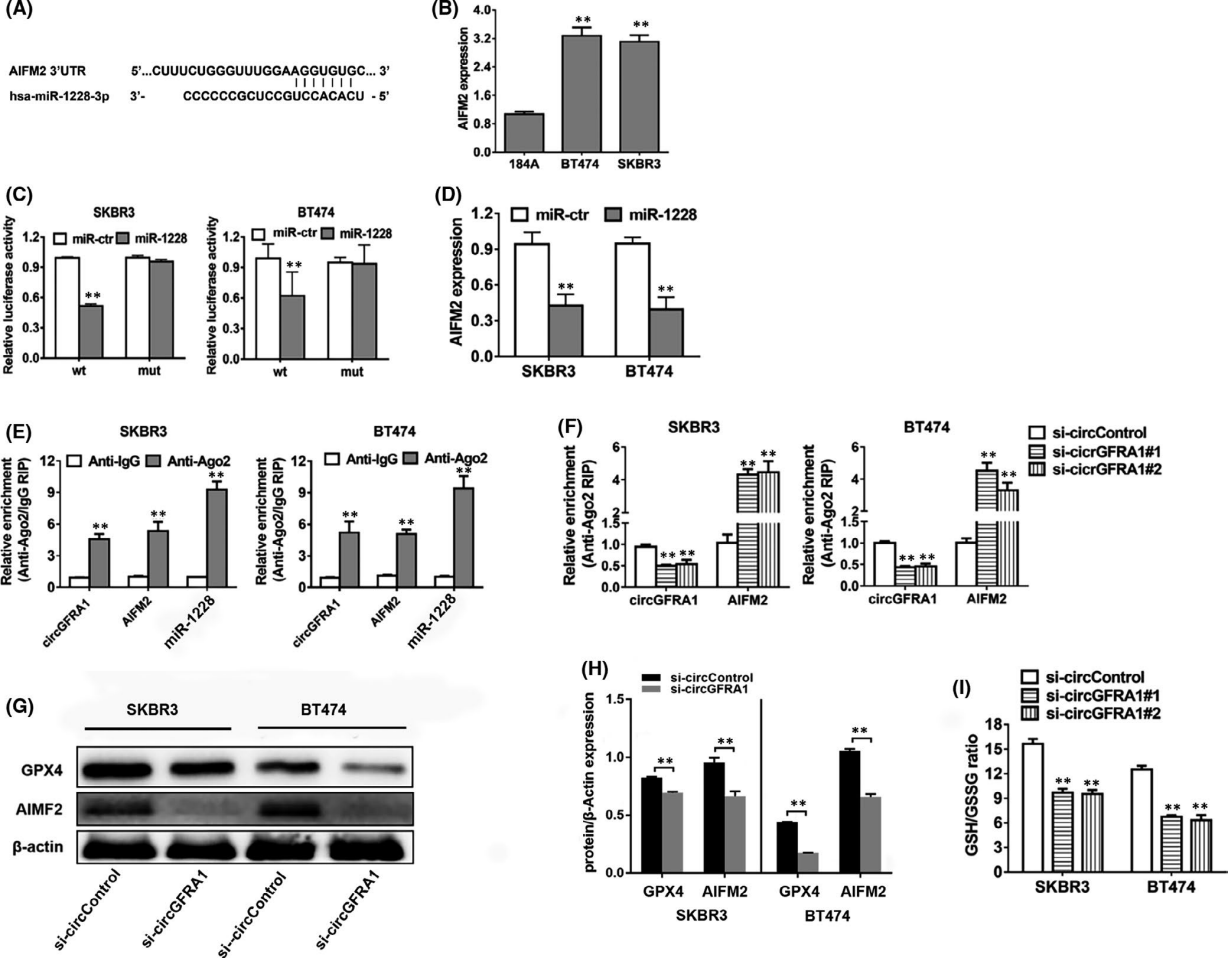
CircGFRA1 silencing enhances ferroptosis in HER‐2‐positive BC. (A) The predicted direct docking sites of miR‐1228 within the AIFM2 sequence. (B) AIFM2 content in the HME (184A) cell line and HER‐2‐positive BC cell lines. (C) The relative luciferase activity of SKBR3 (left) and BT474 (right) cells cotransfected with miR‐1228 mimics, as well as the wild‐type or mutant luciferase reporter of AIFM2. (D) AIFM2 level in cell transfects of miR‐1228 mimics. (E) RIP assay of the relative abundance of circGFRA1, AIFM2 and miR‐1228 in the Ago2 fraction. (F) Cells were transfected, and the relative abundance was determined by an Ago2‐related RIP assay. (G) Western blotting assessment of levels of AIFM2, GPX4 and β‐actin. (H) Quantification and *p* value of Western Blotting. (I) GSH/GSSG Quantification of the GSH/GSSG ratio

The downstream targets of miR‐1228 were predicted with the TargetScan algorithm. The AIFM2 was uncovered target gene of miR‐1228, as illustrated in Figure 5A. To verify the predicted targeted relationship, AIFM2 expression was measured in HER‐2‐positive BC cell lines, and it was demonstrated to be upregulated (Figure 5B). After that, to validate whether miR‐1228 could directly dock to AIFM2 mRNA, a subsequent RIP assay was performed. As a result, the luciferase enzyme activity reduced following insertion with wild‐type luciferase reporter of AIFM2 and miRNA‐1228 mimics, whereas mutant luciferase reporter of AIFM2 lacked this influence (Figure 5C). Besides, miR‐1228 mimics suppressed the expression of AIFM2, indicating that AIFM2 is modulated by miR‐1228 (Figure 5D). Moreover, the Ago2 RIP assay data illustrated that circGFRA1, AIFM2 and miR‐1228 were remarkably abundant in the Ago2 complex (Figure 5E). Downregulation of circGFRA1 remarkably elevated the enrichment of AIFM2 in the Ago2 proportion and diminished circGFRA1 abundance in Ago2 complexes (Figure 5F), illustrating that circGFRA1 competes with AIFM2 in docking to miR‐1228. Next, we determined that with the knockdown of circGFRA1, AIFM2 expression was downregulated in HER‐2‐positive BC cells (Figure 5G). Interestingly, AIFM2 is generally regarded as a ferroptosis suppressor mediated by ubiquinone.^22^ On the basis of the research evidence above, we concluded that silencing circGFRA1 enhances ferroptosis through the circGFRA1‐miR‐1228‐AIFM2 axis. Besides, we discovered that cancer cells with silencing of circGFRA1 displayed a reduction in the glutathione (GSH)/oxidized glutathione (GSSG) ratio and knockout of glutathione peroxidase 4 (GPX4) (Figure 5G,H). Combined with the confirmation that the decrease in the GSH/GSSG ratio results in the deactivation of GPX4, which in turn leads to more toxic lipid reactive oxygen species (ROS) accumulation and ferroptosis induction,^23^, ^24^ we can deduce that knockdown of circGFRA1 promotes ferroptosis. In addition, it has been proven that the two pathways mentioned above act in parallel with each other.^25^ Therefore, knockdown of circGFRA1 promotes ferroptosis in HER‐2‐positive BC via two independent pathways.

## 4 DISCUSSION

Increasing experiments have shown that circRNAs promote or suppress TNBC tumorigenesis.^26^, ^27^ However, it is unclear whether circRNAs has a role in the biological processes of HER‐2‐positive BC. CircGFRA1 is involved in the progress of several cancers.^28^, ^29^ For instance, circGFRA1 is overexpressed and facilitates ovarian cancer development.^28^ CircGFRA1 and miR‐188‐3p cooperatively modulate NSCLC (non‐small cell lung cancer) tumour growth.^29^ Here, we revealed that circGFRA1 was highly upregulated in HER‐2‐positive BC. Furthermore, functional experiments illustrated that repression of circGFRA1 dampened the growth, infiltration and metastasis of HER‐2‐positive BC cells, suggesting that circGFRA1 could regulate BC progression that is HER‐2‐positive. Together, these findings revealed the prognostic and therapeutic value of circGFRA1 in HER‐2‐positive BC.

The ceRNA hypothesis provides insight into the relationships between circRNAs, lncRNAs (long noncoding RNAs), pseudogenes, mRNAs, as ceRNAs crosstalk with or co‐modulate each other through competing for miRNA docking.^30^, ^31^, ^32^ Among ceRNA family members, circRNAs have emerged a research hotspot. Groundbreaking research on the ceRNA role of circRNAs revealed that circRNA CDR1as, which is stable, highly expressed and predominantly cytoplasmic, has the capacity to function as a possible miR‐7 sponge that widely binds to miR‐7 in neuronal tissues.^18^ Herein, we carried out bioinformatics analyses, and the data demonstrated that circGFRA1 harboured an MRE of miR‐1228. Besides, functional assays were used to verify relation of circGFRA1 with miR‐1228. MiR‐1228 has important functions in multiple cancers.^33^, ^34^ Chen et al. uncovered that miR‐1228 is highly expressed in lung cancer there it promotes tumour growth.^33^ Zhang et al. showed that in hepatocellular carcinoma (HCC), miR‐1228 enhances HCC cell proliferation along with metastasis by negatively regulating p53.^34^ This study revealed that circGFRA1 contributes to carcinogenesis in HER‐2‐positive BC by binding to miR‐1228, and the interaction between them plays a significant role in the biological process of HER‐2‐positive BC.

Ferroptosis, an emerging kind of cell death that requires iron and the aggregation of ROS, is considerably distinct from apoptosis, necrosis and autophagy.^35^ Ferroptosis may be restricted by several anti‐ferroptosis systems. The GSH (glutathione)‐GPX4(glutathione peroxidase 4) system is implicated in the suppression of ferroptosis.^36^ In addition, a novel anti‐ferroptosis system, AIFM2 (apoptosis‐inducing factor mitochondrial‐associated 2), also termed as FSP1 (ferroptosis suppressor protein 1), is involved in resistance to ferroptotic cell death. Here, we investigated whether circGFRA1 sponges miR‐1228 to increase AIFM2 levels, which inhibits ferroptosis in HER‐2‐positive BC cells. AIFM2 exerts an inhibitory effect on ferroptosis by using NAD(P)H to reduce CoQ_10_, and reduced CoQ_10_ is an endogenous antioxidant suppressing ferroptosis.^35^, ^37^ AIFM2‐CoQ10‐NAD(P)H and GSH‐GPX4 are two independent pathways that operate in parallel with the anti‐ferroptosis process.^22^ GPX4 is a major enzyme of the GSH‐GPX4 system, and GSH depletion inhibits GPX enzyme activity to decoy ferroptosis.^38^ The outcomes of this study showed that the expression of AIFM2 and GPX4 and the GSH/GSSG ratio were upregulated in HER‐2‐positive BC; therefore, HER‐2‐positive BC cells can activate two pathways to inhibit ferroptosis. This provides a promising therapeutic strategy, as targeting the two pathways above may improve the anticancer therapy of HER‐2‐positive BC.

## 5 CONCLUSION

Herein, we established that circGFRA1 is remarkably upregulated in HER‐2‐positive BC and regulates the malignant behaviour of HER‐2‐positive BC cells. CircGFRA1 is shown to play its regulatory role through its effect on AIFM2 expression by sponging miR‐1228; therefore, the circGFRA1‐miR‐1228‐AIFM2 axis plays a remarkable role in HER‐2‐positive BC tumorigenesis. CircGFRA1 and AIFM2 may serve as promising treatment alternative for HER‐2‐positive BC.

## CONFLICT OF INTEREST

The authors declare that there is no conflict of interest.

## AUTHOR CONTRIBUTION

**Rongfang He:** Funding acquisition (equal); Project administration (equal); Writing‐review & editing (equal). **Bazhabayi Meiheban:** Writing‐original draft (equal). **Xingsheng Qiu:** Project administration (equal); Writing‐review & editing (equal). **Xing Li:** Data curation (equal); Formal analysis (equal); Methodology (equal). **Anli Yang:** Conceptualization (equal); Resources (equal); Software (equal). **Wei Wen:** Data curation (equal); Formal analysis (equal). **Xiaoli Zhang:** Data curation (equal). **Xiangsheng Xiao:** Project administration (equal); Supervision (equal); Validation (equal). **Peng Liu:** Supervision (equal).

## Data Availability

The data that support the fundings of this study are available from the corresponding author upon reasonable request.
